# Annotations

**Published:** 1908-08-01

**Authors:** 


### ANNOTATIONS.

### Tobacco Smoking.

A pamphlet, written by Mr. H. H. Tidswell, a member of our profession, has recently been circulated broadcast. It concerns the evil effects of tobacco smoking, and is an appeal to all and sundry to abandon the habit, and to the medical profession in particular to second the efforts of Mr. Tidswell and the Anti-Narcotic League. Now we would not have it supposed that we favour in the least degree excessive indulgence in tobacco or any other luxury, nor do we shut our eyes to the demonstrated facts that nicotine, or some other constituent of this herb, exerts a most deleterious effect on the musculature of the heart and on certain nerves. But looked at fairly and without bias, the case as presented in this pamphlet is singularly weak. To instance a half-dozen or so of eminent men who do not smoke, and about as many lives of exceptional longevity, backed up by a case in which the onset of phthisis is believed by the author (without evidence) to have been induced by smoking, is to provoke the production of equally inconclusive examples of similar feats by inveterate smokers. To sandwich a considerable number of wholly irrelevant texts from Scripture into the argument seems to us equally beside the point. The truth of the whole matter is probably to be sought somewhere half-way between the positions of the extremists. There is little question that the majority of mankind can enjoy tobacco-smoking in moderation without any obvious harm resulting; and it is equally true that to many, especially brain workers, the habit does bring a certain soothing and beneficent influence. It is also certain that many men misuse the drug by excessive and harmful indulgence in it, to their physical and mental deterioration; and this is particularly true of boys and adolescents, in whom the habit of excessive smoking is a real and pressing evil. Again, there are some whose idiosyncrasy to nicotine is such that they are far better off by total abstinence from tobacco. But, because the latter class exists, it is as hopeful to attempt to root out smoking as it is to abolish tea drinking and alcohol taking for a similar reason.

### The Poisons and Pharmacy Bill.

After many years of struggle the Pharmaceutical Society and the limited companies which carry on the business of druggists seem to have arrived at an understanding which will allow some of the admitted anomalies of the Pharmacy Acts to be remedied by legislation. Those limited companies which describe themselves as "chemists" are each to have at least one qualified person as a director, as well as a qualified manager at each branch shop. Other limited companies and individual druggists with more than one place of business are also to have qualified managers for branch shops, and the names of such managers are to be conspicuously exhibited in some part of the premises. Companies are to become subject to the poison regulations in the same way as individual druggists. Lord Crewe's Poisons and Pharmacy Bill, which now embodies the salient features of the compromise, has passed through its most critical stages in the House of Lords, and may pass the House of Commons this year. In the Lower House it will be closely watched by some of the medical members, notably Sir John Batty Tuke, who made several ineffectual attempts, as a member of the Joint Select Committee on the Bill, to bring the measure into line with the expert opinion of the medical profession. The Bill still contains provisions of doubtful wisdom. Thus, while a new and improved schedule of poisons has been inserted, and some restrictions as to labelling are to be placed on the sale of strong mineral acids, of which the retail distribution is at present unrestrained, the Government proposes to give County Councils powers to grant licenses to persons other than qualified druggists for the sale of poisonous preparations for use in horticulture. This involves the creation of a precedent which is probably unnecessary and certainly likely to encourage those grocers who wish to obtain powers to sell packed proprietary medicines containing poison. The principle of the existing law is that the vendor of poisons must, in the interests of public safety, have shown himself to possess a competent practical knowledge of chemistry and pharmacy. This is a salutary principle which should be firmly adhered to so far as concerns all articles alleged to possess medicinal properties.

### "The Storage of River Water."

At a meeting of the Metropolitan Water Board, held on July 17, the Water Examination Committee presented a first report on research work by Dr. A. C. Houston, director of Water Examinations, Metropolitan Water Board, on the vitality of the typhoid bacillus in artificially infected samples of raw Thames, Lea, and New River water, with special reference to the question of storage. Dr. Houston says:—It is possible to show that under storage a "change" has occurred in river water, which in all probability can only have resulted from sustained storage, and that this "change" is associated with relative (perhaps even absolute) "safety" of the water so far as the typhoid fever bacillus is concerned. The advantages accruing from even a few days' storage may be so material that, exceptional cases apart, the use of raw unstored river water for filtration purposes should strongly be deprecated. It is not impossible that the additional "safety" conferred by adequate storage may come to be regarded as a reasonable pretext for filtration through mechanical filters, at specially rapid rates, thereby effecting considerable economies in the cost of filtration, as ordinarily practised; but any departure from old-established filtration custom should not be entertained in the absence of convincing experimental proof of the reliability of the new process. During a considerable part of each year, there is an abundance of water of relatively good quality in the Thames and Lea, and the existing storage reservoirs are sufficiently large, in the aggregate, to improve enormously the water derived from these rivers. The advantages accruing from adequate storage of water are of a general character and are not limited to the elimination of danger from typhoid fever.

